# Progress and challenges of implantable neural interfaces based on nature-derived materials

**DOI:** 10.1186/s42234-021-00067-7

**Published:** 2021-04-27

**Authors:** Eugenio Redolfi Riva, Silvestro Micera

**Affiliations:** 1 The BioRobotics Institute and Department of Excellence in Robotics and AI, Scuola Superiore Sant’Anna, Pisa, Italy; 2 Bertarelli Foundation Chair in Translational Neuroengineering, Centre for Neuroprosthetics and Institute of Bioengineering, School of Engineering, École Polytechnique Fédérale de Lausanne (EPFL), Lausanne, Switzerland

**Keywords:** Nature-derived materials, Implantable neural Interface, Biocompatibility, Long-term implant, Coating, Insulation material

## Abstract

Neural interfaces are bioelectronic devices capable of stimulating a population of neurons or nerve fascicles and recording electrical signals in a specific area. Despite their success in restoring sensory-motor functions in people with disabilities, their long-term exploitation is still limited by poor biocompatibility, mechanical mismatch between the device and neural tissue and the risk of a chronic inflammatory response upon implantation.

In this context, the use of nature-derived materials can help address these issues. Examples of these materials, such as extracellular matrix proteins, peptides, lipids and polysaccharides, have been employed for decades in biomedical science. Their excellent biocompatibility, biodegradability in the absence of toxic compound release, physiochemical properties that are similar to those of human tissues and reduced immunogenicity make them outstanding candidates to improve neural interface biocompatibility and long-term implantation safety. The objective of this review is to highlight progress and challenges concerning the impact of nature-derived materials on neural interface design. The use of these materials as biocompatible coatings and as building blocks of insulation materials for use in implantable neural interfaces is discussed. Moreover, future perspectives are presented to show the increasingly important uses of these materials for neural interface fabrication and their possible use for other applications in the framework of neural engineering.

## Background

For decades, science fiction literature has triggered human imagination and curiosity on the creation of devices able to communicate with the nervous system and capable of restoring lost cognitive and sensory-motor functionalities (Cutrone and Micera 2019). This literary fascination has turned into reality because of the emergence of micro-nanotechnologies, which paved the way for the manufacture of devices that act as interfaces between the biological (neurons and nerves) and artificial worlds (computers, artificial limbs, etc.) (Fekete and Pongrácz 2017; Wang et al. 2018). A neural interface (NI) is a bioelectronic device capable of stimulating a population of neurons or nerve fascicles and recording electrical signals in a specific area, with the aim of restoring physiological neural activity and re-establishing sensory-motor feedback through prosthetic devices (del Valle and Navarro 2013; Rijnbeek et al. 2018). NIs are categorized into three main classes: cortical, spinal cord and peripheral implants. An NI consists of an insulating material with specific geometric features that is able to interact with a designated tissue area and one or more conductive materials that carry recorded or stimulating electrical signals (Bettinger et al. 2020; Jastrzebska-perfect et al. 2020; Rivnay et al. 2017; Rochford et al. 2019).

Despite the success of NI in restoring sensory-motor functions, poor biocompatibility of these devices impedes long-term usage of NIs (Lacour et al. 2016; Wurth et al. 2017). This incompatibility is caused by the NI implantation process itself, which requires penetration of the nervous tissues with a rigid probe, but its long-term effects depend on the properties of the NI material. Traditional insulating material (silicon, polyimide and parylene C) and conducting material (gold, titanium, aluminum, iridium oxide and platinum) used in NI fabrication possess completely different structural and physiochemical properties with respect to the tissue with which they must interface. The mechanical mismatch (E_polyimide_ ≈ 2.5 GPa (Rousche et al. 2001), E_brain_ ≈ 5.51 kPa (Subbaroyan et al. 2005), E_tibial nerve rabbit_ ≈ 500 kPa (Kwan et al. 1992)), the different chemical structures, and different physical properties and geometries between NI components and neural tissue activate the host immune system, triggering an inflammation process called foreign body reaction (FBR) (Lotti et al. 2017; Renz et al. 2018). An ideal NI exhibits stable electrical performance to allow selectivity of the recorded/stimulating signal, and the fabrication materials should match the physiochemical and mechanical properties of the surrounding tissues, thus allowing tissue-implant integration. However, upon NI implantation, FBR triggers acute and subsequent chronic inflammatory responses at the interface with neurons and nerves, damaging surrounding tissues and worsening NI functionality (de la Oliva et al. 2018). Recording performances have been demonstrated to decrease drastically approximately 1 month after electrode implantation, and electrical impedance at the tissue/device interface increases as a consequence of fibrotic tissue formation around the implant (Gunasekera et al. 2015; Karumbaiah et al. 2013). Moreover, immune cells such as macrophages continue to move to the site of the implant, releasing inflammatory cytokines that sustain the immune response and compromising the long-term usability of the NIs (Del Valle et al. 2015).

In this context, the use of nature-derived materials (NMs) for NIs can pave the way for consistent improvements in NIs long-term implantation feasibility (Chen and Allen 2012). NMs such as extracellular matrix (ECM) components, proteins and polysaccharides have been employed for decades in biomedical science (Boddohi and Kipper 2010; Chow et al. 2008; Macaya and Spector 2012; Muskovich and Bettinger 2012). The objective of this review is to highlight the progress and challenges concerning the impact of NMs in the framework of implantable NIs. In particular, the contributions of NMs is discussed in two sections, one describing their use as biocompatible coatings and another describing their use as building blocks of NIs to improve electrode long-term safety. Finally, future perspectives are addressed to show the progressive replacement of traditional NIs fabrication materials and NMs use in other fields of neural engineering, such as in the development of biodegradable neural interfaces.

## State of the art

With chemistry supplying almost unlimited types of materials, NMs are never-ending sources of inspiration that provide substances with remarkable properties for devices used in biomedical science (Chow et al. 2008; Muskovich and Bettinger 2012; Yu et al. 2018). NMs such as polysaccharides (Boddohi and Kipper 2010; Fujie et al. 2009; Redolfi Riva et al. 2013, 2014, 2017), nucleic acids (Lissek 2017; Wiraja et al. 2019), proteins (Parenteau-Bareil et al. 2010), peptides (Lee and Lee 2017) and lipids have been used for decades for the fabrication of biomedical devices and nanostructure materials. Compared to synthetic materials, NMs are advantageous because of their outstanding biocompatibility, degradation without inducing cytotoxicity or immunogenic release of compounds and physiochemical properties that are similar to those of biological tissue (Macaya and Spector 2012; Pradhan et al. 2020). For example, ECM components such as collagen and hyaluronan possess biochemical cues that enhance cell adhesion and proliferation (Hussey et al. 2018). Moreover, polysaccharides such as cellulose, chitosan, alginate, dextran and agarose are very interesting examples of NMs since they possess rheological properties similar to those of ECM glycosaminoglycans. Several contributions of NMs in the framework of implantable NIs have been published and can be categorized into two main topics regarding their use: biocompatible coatings and building blocks of NIs.

### Nature-derived materials as biocompatible coatings of neural interfaces

#### Nanostructured coatings

**Fig. 1:**
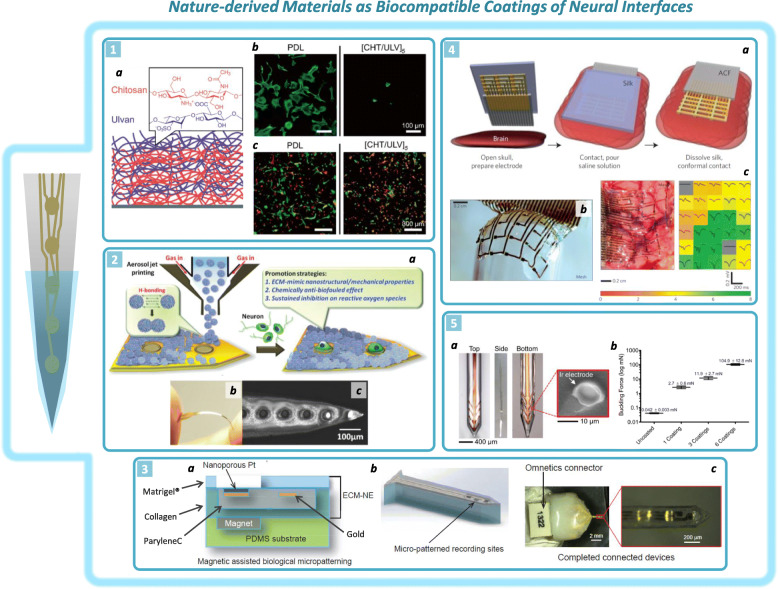
Examples of NMs as biocompatible coatings of NIs. 1: (a) *Layer-by-layer* deposition of chitosan and ulvan multilayers on silicon surfaces. (b) Confocal microscopy (CM) images showing astrocyte preferential adhesion on a poly-D-lysine surface with respect to a multilayer surface. (c) CM images of neurons (red) and astrocytes (green) cocultured on poly-D-lysine and multilayer surfaces. Multilayer surfaces enhance neuron adhesion and reduce astrocyte anchorage (Reproduced and adapted with permission from [Moon et al. 2020] Copyright 2020, ACS Publications). 2: (a) Schematic illustration of amphiphilic siloxane-modified chitosan nanogel patterned on a polyimide-based NI surface. The nanogel is loaded with oligo-proanthocyanidin and provides EMC-mimicking behavior. (b) Photograph and SEM image (c) of the device showing nanogel patterned onto polyimide, exposing electrode active sites (Reproduced and adapted with permission from [Huang et al. 2015] © 2015 *John Wiley & Sons*, Inc.). 3: (a) Schematic illustration of the EMC-based intracortical electrode in the side view and after laser ablation of the electrode (b). Image of the total device with the electrode tip in the inset (c) (Reproduced and adapted with permission from [Shen et al. 2015] Copyright 2015, Nature.). 4: (a) Schematic illustration of the process of brain tissue adhesion of a cortical electrode array caused by silk supporting layer dissolution. (b) Picture of the same electrode adhering onto a glass hemisphere during silk layer dissolution. (c) Electrode array perfectly adhered to an animal visual cortex during recording activity (left) and the color map of the average evoked response from each electrode (right) showing the rms amplitude of the recorded signal at each electrode active site (Reproduced and adapted with permission from [Kim et al. 2010] Copyright 2010, Nature). 5: (a) Polyimide-based electrode coated with a silk layer on its bottom side, exposing the surface of the recording active sites. (b) Average buckling force levels of uncoated and silk-coated electrodes prepared with 1, 3 and 6 coating steps (Reproduced and adapted with permission from [Tien et al. 2013] © 2013 *John Wiley & Sons*, Inc.)

Biocompatible coatings for NIs (Fig. 1) have been shown to be promising solutions to reduce tissue inflammation and scar tissue formation upon NI implantation, thus enhancing their long-term safety and stability (Woods et al. 2020). The idea is to functionalize the electrode surface with a *buffer layer*, such as a hydrogel (Yuk et al. 2019), at the tissue/implant interface that is able to reduce the adhesion of microglia, fibroblasts and macrophages at the implant surface, thus reducing scar tissue formation around the implant (Cutrone and Micera 2019; Mohan et al. 2015; Wellman et al. 2018; Zhang and Chiao 2015). Ideally, a coating provides cytocompatible anchorage for neuronal cells. In this regard, the pioneering work of Ravi Bellamkonda concerning nanoscale coating of silicon surfaces for NIs is notable (He et al. 2006). The *layer-by-layer* technique has been used because of its remarkable versatility advantageous for obtaining nanostructure coatings with tunable thickness, surface roughness, suitable Young’s modulus and swelling capacity (Silva et al. 2016; Zhang et al. n.d.). In this study, polyethyleneimine (PEI), gelatin and chitosan were used, and the absorption capability of laminin inside the nanostructure polymer network was studied. The results confirmed enhanced neuronal adhesion and axon sprouting with respect to the bare silicon substrate. Recently, a *layer-by-layer* technique was proposed for use in coating silicon surfaces with marine polysaccharides, including chitosan, derived from crustacean shells, and ulvan, isolated from green algae, which have physiochemical properties similar to those of glycosaminoglycans, thus providing a convenient ECM-like environment for neural cell adhesion (Moon et al. 2020). The results showed enhanced hippocampal neuron proliferation and reduced astrocyte adhesion on the NM-based coating, suggesting its use to improve cortical electrode biocompatibility. An interesting example in the framework of NIs coating was proposed by Righi and colleagues, who suggested using IKV peptide-functionalized polyimide, which showed enhanced PC12 cell adhesion and neurite outgrowth (Righi et al. 2018).

#### Silk-based and ECM-like microstructure coatings

Fibroin derived from silk is another NM that has inspired multiple studies in the context of NIs because of its excellent biocompatibility and mechanical properties (Kundu et al. 2013). Fibroin is extracted from *Bombyx mori* cocoons and has been widely used in different frameworks of neural engineering, such as biodegradable stiffeners to improve electrode tissue penetration, biocompatible coatings and dissolvable sacrificial layers (Kim et al. 2010; Lecomte et al. 2015; Metallo and Trimmer 2015; Tang-Schomer et al. 2014; Tien et al. 2013). Notably, Rogers and colleagues described a clever way to exploit silk film as a supporting layer to improve NI conformability with target brain tissue (Kim et al. 2010). Successful transfer of a planar cortical NI on the feline brain demonstrated an excellent level of probe adhesion to the tissue, as ensured by fibroin layer dissolution. This process guaranteed good recording performance, as shown in animal experiments.

Moreover, NMs coating of neural interfaces has also been envisioned for fabricating multifunctional NIs with increased electrical performance and drug release functionality, as demonstrated by Abidian and Martin (2009); in their work, an alginate hydrogel was fabricated on an electrode surface previously coated with dexamethasone (DEX)-loaded PLGA nanofibers. Alginate was exploited for subsequent electrodeposition of PEDOT to enhance electrical performance. Furthermore, alginate hydrogels have also been used to slow DEX diffusion by approximately 50% compared to uncoated electrodes. NMs can also be used as active molecules to functionalize NIs for anti-inflammatory purposes. Natural oligo-proanthocyanidin with antioxidizing properties has been incorporated into amphiphilic siloxane-modified chitosan nanoparticles. This nanogel has been deposited onto polyimide-based NIs to provide a drug-releasing coating with ECM-mimicking nanostructure behavior (Huang et al. 2015).

Other studies where considerable effort has been made to modify traditional microfabrication techniques to integrate NMs in electrode fabrication using ECM-like coatings are also worthy of mention (Chen et al. 2017; Shen et al. 2015; Vitale et al. 2018). ParyleneC was embedded in a type I collagen layer, and magnetic-assisted micropatterning was used to coat the electrode surface with a Matrigel mixture, exposing electrode active sites for neural recording. The electrode showed improved biocompatibility, as reported for in vivo implantation (Shen et al. 2015). However, the authors reported that the thickness of the EMC-like structure and consistent swelling of the device after implantation may be potentially dangerous to the neuronal structure and can diminish recording capability.

All the cited studies demonstrated the remarkable contribution that NMs can make to the framework of neural engineering. Although its ability to reduce FBR has been demonstrated in multiple studies, coating stability over time is still a subject of debate for some critical reasons (Wellman et al. 2018). Macroscopic hydrogel coatings, such as ECM-like structures, suffer from instability over time because of the oxidation process and dimensions compared with electrode thickness. These issues can cause progressive coating detachment from probe surfaces during implantation and increase electrical impedance over time. Another problem of macroscopic hydrogel coatings is consistent swelling upon implantation (Goding et al. 2019). In this regard, highly hydrophilic materials, including NMs, undergo consistent water uptake with swelling ratios that can be more than double their dry size (Catoira et al. 2019; Marcombe et al. 2019). This process can diminish the recording capability by increasing the distance between the electrode active site and neurons and can also lead to progressive detachment of the electrode conductive layer.

For these reasons, we believe that different NIs coatings made from nanostructure materials, such as *layer-by-layer* nanocoating and peptide functionalization, are preferable solutions to enhance biocompatibility for achieving better electrode tissue integration and electric performance (Olczak et al. 2019).

### Nature-derived materials as building blocks of neural interfaces

**Fig. 2:**
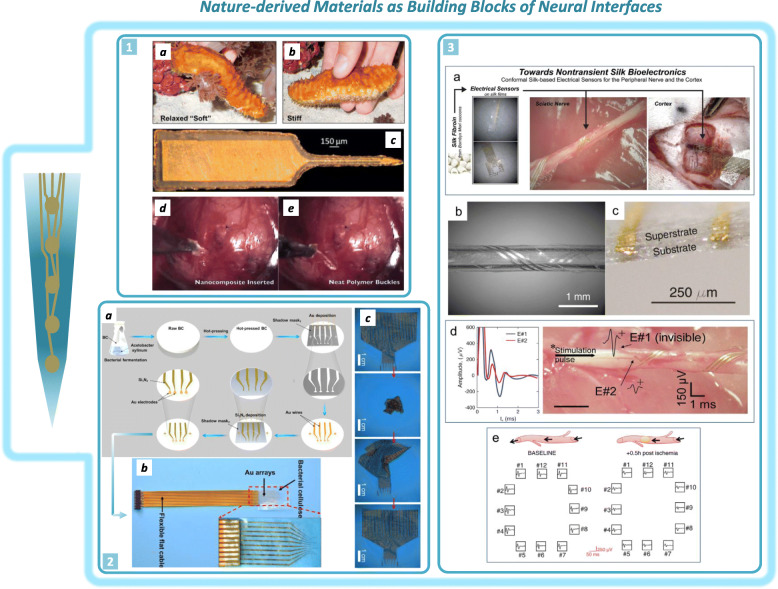
Examples of NMs as building blocks of NIs. 1: Picture of a sea cucumber changing its stiffness from a soft (a) to a stiff (b) configuration. (c) Laser-micromachined cortical probe with a parylene C capping layer fabricated with a 12.2% v/v poly (vinyl acetate) (PVAc)/cellulose nanofiber nanocomposite used as a mechanically adaptive substrate (thickness: 60 μm). Photographs of an electrode during its insertion into the brain of a rat showing correct penetration of the nanocomposite electrode (d) and the buckling effect of a neat polymer electrode used as a control (e), demonstrating that cellulose nanofibers can effectively act as stiffness-adapting elements (Reproduced and adapted with permission. [Harris et al. 2011d,e; Capadona et al. 2012 a,b,c] Copyright 2011, IOP Publishing; Copyright 2012, Springer). 2: (a) Scheme of the fabrication steps of a bacterial cellulose-based NI. (b) Photograph of a bacterial cellulose-based device connected to a recording system. (c) Images showing the extreme flexibility of a bacterial cellulose-based NI, which remains intact after repeated twisting and untwisting (100 times) (Reproduced and adapted with permission from [Yang et al. 2018] Copyright 2018, ACS Publications). 3: (a) Schematic illustration describing the use of silk/fibroin as a substrate for the nontransient neural interface for the peripheral nerve and for the cortex. (b) Silk-based NI wrapped around a silicon tube. (c) Picture showing the electrode silk substrate and superstrate with embedded conductive lines. (d) Silk-based NI wrapped around the sciatic nerve (right) to record the electrical signal at two different points (left). (e) Comparative cortical activity recorded by silk-based cortical electrodes prior to (left) and post (right) ischemia induction (Reproduced with permission from [Patil et al. 2020a, b] © 2020 *John Wiley & Sons,* Inc.)

A promising use of NMs in the framework of implantable NIs is as electrode building blocks (Fig. 2). Given the properties of natural insulators, the use of NMs can also be imagined for the fabrication of structural/insulation layer of NIs. In this view, a new solution to electrode fabrication may pave the way for consistent innovation in NIs design. Indeed, we believe that the contribution of NMs in this context could change the paradigm of flexible NIs fabricated with synthetic insulation materials.

The inspiring work of the Jeffrey Capadona group is pioneered the use of NMs as building blocks of the insulation material used for NIs (Capadona et al. 2008). Hybrid natural/synthetic flexible materials have been used to reduce the chronic immune response, enhancing the long-term stability of implanted NIs (Capadona et al. 2012; Harris et al. 2011). Inspired by the sea urchin behavior of altered stiffness, a biomimetic approach has been used to develop a stimuli-responsive intracortical electrode formed by cellulose nanowhisker-doped (TC-doped) polyvinyl acetate (PVAc) (Capadona et al. 2008; Shanmuganathan et al. 2010); this material possesses outstanding switchable mechanical properties as shown upon water absorption, when the electrode undergoes drastic softening, with the Young’s modulus changing from 3420 ± 98 MPa (dry state) to 22 ± 7 MPa (swollen state) (Hess et al. 2011). This switching ability was exploited to insert this electrode inside the brain, taking advantage of its rigidity in the dry state. Enhanced integration with biological tissue has been demonstrated by in vitro and in vivo investigations that showed reduced chronic inflammation over time (Nguyen et al. 2014). Although cellulose is the basic structural polysaccharide of plants, it is also produced by bacteria such as *Acetobacter xylinum* in the form of bacterial cellulose (BC), which has higher mechanical strength than plant-derived cellulose (Esa et al. 2014). BC has recently been used as an insulation layer of cortical electrodes after being processed into thin films by hot pressing (Yang et al. 2018). After further microfabrication steps, conductive layers were deposited onto the BC insulation layer to produce the final electrode. This BC-based device has superior advantages compared to traditional insulation materials, with mechanical properties similar to brain tissue and extreme conformability to brain tissue because of a bending stiffness that is 1/5200 that of polyimide-based electrodes (Yang et al. 2018).

In another interesting work in the context of NMs as building blocks of NIs, nontransient silk electrodes were used for neural recording (Patil et al. 2020a, b). In this study, a smart process used to modify the traditional microfabrication technique conferred NM with adaptability for integration, with silk used as a water-stable insulating layer. Water annealing was used to achieve a water-stable nontransient silk NI, and subsequent conductive layer deposition led to the formation of a flexible silk electrode. Experiments on material stability in physiological environments and animal tests illustrated the remarkable potential of this electrode as a sensing interface for neural signal recording in either the cortex or peripheral nervous system (Patil et al. 2020a, b). This type of nontransient NM-based electrode and the great potential of NMs to the pursuit of the biocompatibility enhancement could represent a turning point in NIs long term reliability. This change in perspective can pave the way for the development of highly conformable and tough NM-based electrodes whose long-term performances can be better than those of traditional NIs because of the abovementioned advantages of NMs over traditional synthetic materials. We believe that this vision can be a source of inspiration for scientists to adapt current microfabrication techniques to employ NMs in the fabrication chain of NIs with the goal of progressively replacing traditional NIs building blocks such as resins and elastomers.

## Challenges and future perspectives

### Promising strategies for NMs integration in neural interface design

Despite the aforementioned promising uses of NMs, poor mechanical properties, consistent swelling upon water uptake and instability are the main problems that can have a negative impact on NIs performance. These problems can be overcome by polymer crosslinking, annealing treatments or chemical modification and by using alternative fabrication strategies. In the framework of NIs coating, the *layer-by-layer* technique is an advantageous and versatile strategy, ensuring good electrode/tissue integration and reducing FBR effects over time. Moreover, the encapsulation of drugs, conductive elements and functional nanoparticles inside this structure allows the imagining of a new coating concept: a smart nanostructure layer with improved compatibility for neurons, increased electrical performance and sustained release of active molecules over time. This new class of NMs-based coatings can strongly impact the long-term stability of an NI, avoiding the need for electrode explants. Hence, future directions for the use of NMs can be imagined in this type of framework, where integration with other functional elements can be the key for the development of a new NI concept. In fact, the use of NMs for electrode insulating layer fabrication can efficiently impact NI design, with the objective of enhancing electrode tissue integration. In our view, the abovementioned works on nontransient silk (Patil et al. 2020a, b) and cellulose-based electrodes (Yang et al. 2018) represent promising and challenging new lines of research with significant potential to enhance the long-term performance and tissue integration of NIs.

### Nature-derived conductive polymers: inspiring solutions for bioelectronic devices in neural engineering

Conductive polymers have been widely used in neural engineering for decades as alternatives to metallic structures for the design of conductive layers of biomedical devices. George G. Malliaras’ work on organic transistors for brain activity recording using poly (3,4-ethylenedioxythiophene) doped with polystyrene sulfonate (PEDOT:PSS) as a conductive element is worthy of citation (Khodagholy et al. 2013). Another interesting study reported the use of polypyrrole (PPy) as a conductive polymer for silk-based scaffolds used in neural tissue engineering (Zhao et al. 2018). The most noteworthy examples of electroconductive polymers used for organic electronics are synthetic in nature; however, a detailed discussion of their roles in neural engineering is beyond the scope of this paper. The recent review of Rylie Green and Mohammad Reza Abidian offers a more comprehensive presentation on this class of materials (Green and Abidian 2015). Nevertheless, in recent years, novel examples of electroconductive polymers inspired by nature-derived materials have been explored for use in organic electronics. In this regard, natural compounds such as eumelanin, a protein derived from the oxidative polymerization of 5,6-dihydroxyindoles and used as UV-protection molecules, have already been used to enhance photocurrent production in porous silicon-based optoelectronic devices (Antidormi et al. 2018). In the framework of neural engineering, spin-coated melanin films have been reported to support and enhance PC12 growth and neurite sprouting (Bettinger et al. 2009). Hence, the semiconducting properties of eumelanin (D’Ischia et al. 2009) coupled to its processability with the microfabrication technique suggest eumelanin as a suitable material for the conductive layer of NIs. Other natural compounds that may be used in this framework include carotenoids and pigments such as indigo. Beta-carotene, a red-orange pigment known for its antioxidizing properties (Sies et al. 1992), displays electroactive properties since it exhibits p-type field effect semiconducting behavior (Burch et al. 2004). For this reason, it has been used for organic electronic devices such as solar cells (Yakuphanoglu et al. 2006). Furthermore, indigo, a natural pigment produced by *Indigofera tinctoria,* has been proposed for use in organic field effect transistors upon deposition through thermal evaporation (Irimia-Vladu et al. 2012). All these studies reveal the substantial contribution that NMs may make to the framework of implantable NIs because of their remarkable properties, allowing us to imagine future development of electrodes comprised entirely of NMs. These new NIs may represent a turning point for future electrode fabrication in neural engineering, envisioning low-cost NIs with excellent tissue/electrode integration for long-term implantation.

### Envisioning NMs use in novel biodegradable electronic devices

Biodegradable electronics for the stimulation/recording of neural signals are other interesting products where NMs use can be envisioned, particularly because of the degradation process of NMs upon contact with biological media (Feig et al. 2018; Irimia-Vladu 2014; Nair and Laurencin 2007). As reported above, NI implantation triggers a chronic inflammatory response, and a second invasive surgery to remove the electrode is required to stop this response. In this framework, electrical stimulation and recording may be useful only in a certain therapeutic window, depending on the pathology to be treated and the time scale of the event to be recorded. A biodegradable NI may be a promising and clever solution for the treatment of neurological disorders, such as epilepsy, for deep brain stimulation and for recording neural signals (Shan et al. 2019). In the past few years, scientists have mainly focused on synthetic materials for biodegradable NI fabrication (Li et al. 2018). A recent work discussing biodegradable NIs for recording stimulus-evoked activity and spontaneous activity in the auditory cortex is worthy of citation (Zhang et al. 2020). Poly (glycerol sebacate), a synthetic material created from mammalian metabolites glycerol and sebacic acid, has been used as a biodegradable insulating layer, and magnesium has been used as a biodegradable conductive material because of its good electrical properties (Johnson and Liu 2013; Sebaa et al. 2013).

The exploitation of NMs can also have a significant impact in this field, although very few studies have reported on this topic. Considerable examples in this context are the studies describing fibroin as a biodegradable substrate for biosensors and transient electronic circuits described in the recent review of Patil and colleagues (Patil et al. 2020a, b) that show how the scientific community is starting to investigate the use of NMs for the realization of biodegradable NIs. To achieve the goal of NM use in biodegradable NIs, more effort will be required to adapt microfabrication techniques to the implementation of natural compounds in the process chain of flexible NIs to allow an ever greater incorporation of NMs into their structure.

## Conclusions

This review highlights how the framework of implantable NIs may benefit from the integration of NMs in the fabrication process. Especially when processed through the *layer-by-layer* technique, NMs have shown good cytocompatibility towards neurons and the possibility to be processed into nanostructured coatings to improve electrode biocompatibility and to provide additional capabilities, such as improved electrical performance and sustained drug release over time. Furthermore, remarkable contributions of NMs have been shown when natural compounds have been used as building blocks of neural interfaces to reduce mechanical mismatch at the electrode/tissue interface. The remarkable properties of NMs can also be envisioned for use in fabricating new biodegradable neural electrodes to treat neurological disease by implanting the probe into the brain without requiring a second surgery to remove it.

In conclusion, we believe that in all the analyzed frameworks, the excellent advantages of NM over synthetic materials can offer considerable benefit in terms of enhancing NIs biocompatibility for long-term implants.

## Data Availability

Not applicable.

## Abbreviations

NMs: Nature-derived materials
NIs: Neural interfaces
ECM: Extracellular matrix
FBR: Foreign body reaction

## References

[CR1] Abidian MR, Martin DC (2009). Multifunctional nanobiomaterials for neural interfaces. Adv Funct Mater.

[CR2] Antidormi A, Aprile G, Cappellini G, Cara E, Cardia R, Colombo L, Farris R, D’Ischia M, Mehrabanian M, Melis C, Mula G, Pezzella A, Pinna E, Redolfi Riva E (2018). Physical and chemical control of Interface stability in porous Si-Eumelanin hybrids. J Phys Chem C.

[CR3] Bettinger CJ, Bruggeman JP, Misra A, Borenstein JT, Langer R (2009). Biocompatibility of biodegradable semiconducting melanin films for nerve tissue engineering. Biomaterials.

[CR4] Bettinger CJ, Ecker M, Daniel T, Kozai Y, Malliaras GG, Meng E, Voit W (2020). Recent advances in neural interfaces — materials chemistry to clinical translation.

[CR5] Boddohi S, Kipper MJ (2010). Engineering nanoassemblies of polysaccharides. Adv Mater.

[CR6] Burch RR, Dong YH, Fincher C, Goldfinger M, Rouviere PE (2004). Electrical properties of polyunsaturated natural products: field effect mobility of carotenoid polyenes. Synth Met.

[CR7] Capadona JR, Shanmuganathan K, Tyler DJ, Rowan SJ, Weder C (2008). Stimuli-responsive polymer nanocomposites inspired by the sea cucumber dermis. Science.

[CR8] Capadona JR, Tyler DJ, Zorman CA, Rowan SJ, Weder C (2012). Mechanically adaptive nanocomposites for neural interfacing. MRS Bull.

[CR9] Catoira MC, Fusaro L, Di Francesco D, Ramella M, Boccafoschi F (2019). Overview of natural hydrogels for regenerative medicine applications. J Mater Sci Mater Med.

[CR10] Chen N, Tian L, Patil AC, Peng S, Yang IH, Thakor NV, Ramakrishna S (2017). Neural interfaces engineered via micro- and nanostructured coatings. Nano Today.

[CR11] Chen S, Allen MG (2012). Extracellular matrix-based materials for neural interfacing. MRS Bull.

[CR12] Chow D, Nunalee ML, Lim DW, Simnick AJ, Chilkoti A (2008). Peptide-based biopolymers in biomedicine and biotechnology. Mater Sci Eng R Rep.

[CR13] Cutrone A, Micera S (2019). Implantable neural interfaces and wearable tactile systems for bidirectional neuroprosthetics systems. Adv Healthc Mater.

[CR14] D’Ischia M, Napolitano A, Pezzella A, Meredith P, Sarna T (2009). Chemical and structural diversity in eumelanins: unexplored bio-optoelectronic materials. Angew Chem Int Ed.

[CR15] de la Oliva N, Navarro X, del Valle J (2018). Time course study of long-term biocompatibility and foreign body reaction to intraneural polyimide-based implants. J Biomed Mater Res A.

[CR16] Del Valle J, De La Oliva N, Müller M, Stieglitz T, Navarro X (2015). Biocompatibility evaluation of parylene C and polyimide as substrates for peripheral nerve interfaces. Int. IEEE/EMBS Conf. Neural Eng. NER 2015.

[CR17] del Valle J, Navarro X (2013). Interfaces with the peripheral nerve for the control of neuroprostheses. Int Rev Neurobiol.

[CR18] Esa F, Tasirin SM, Rahman NA (2014). Overview of bacterial cellulose production and application. Agric Agric Sci Procedia.

[CR19] Feig VR, Tran H, Bao Z (2018). Biodegradable polymeric materials in degradable electronic devices. ACS Cent Sci.

[CR20] Fekete Z, Pongrácz A (2017). Multifunctional soft implants to monitor and control neural activity in the central and peripheral nervous system: a review. Sensors Actuators B Chem.

[CR22] Fujie T, Matsutani N, Kinoshita M, Okamura Y, Saito A, Takeoka S (2009). Adhesive, flexible, and robust polysaccharide nanosheets integrated for tissue-defect repair. Adv Funct Mater.

[CR23] Goding J, Vallejo-Giraldo C, Syed O, Green R (2019). Considerations for hydrogel applications to neural bioelectronics. J Mater Chem B.

[CR24] Green R, Abidian MR (2015). Conducting polymers for neural prosthetic and neural Interface applications. Adv Mater.

[CR25] Gunasekera B, Saxena T, Bellamkonda R, Karumbaiah L (2015). Intracortical recording interfaces: current challenges to chronic recording function. ACS Chem Neurosci.

[CR26] Harris JP, Hess AE, Rowan SJ, Weder C, Zorman CA, Tyler DJ, et al. In vivo deployment of mechanically adaptive nanocomposites for intracortical microelectrodes. J Neural Eng. 2011;8(4). 10.1088/1741-2560/8/4/046010. 10.1088/1741-2560/8/4/046010 PMC4134134 21654037

[CR27] He W, McConnell GC, Bellamkonda RV (2006). Nanoscale laminin coating modulates cortical scarring response around implanted silicon microelectrode arrays. J Neural Eng.

[CR28] Hess AE, Capadona JR, Shanmuganathan K, Hsu L, Rowan SJ, Weder C, Tyler DJ, Zorman CA (2011). Development of a stimuli-responsive polymer nanocomposite toward biologically optimized, MEMS-based neural probes. J Micromech Microeng.

[CR29] Huang WC, Lai HY, Kuo LW, Liao CH, Chang PH, Liu TC, Chen SY, Chen YY (2015). Multifunctional 3D patternable drug-embedded nanocarrier-based interfaces to enhance signal recording and reduce neuron degeneration in neural implantation. Adv Mater.

[CR30] Hussey GS, Dziki JL, Badylak SF (2018). Extracellular matrix-based materials for regenerative medicine. Nat Rev Mater.

[CR31] Irimia-Vladu M (2014). “Green” electronics: biodegradable and biocompatible materials and devices for sustainable future. Chem Soc Rev.

[CR32] Irimia-Vladu M, Gåowacki ED, Troshin PA, Schwabegger G, Leonat L, Susarova DK, Krystal O, Ullah M, Kanbur Y, Bodea MA, Razumov VF, Sitter H, Bauer S, Sariciftci NS (2012). Indigo - a natural pigment for high performance ambipolar organic field effect transistors and circuits. Adv Mater.

[CR33] Jastrzebska-perfect P, Chowdhury S, Spyropoulos GD, Zhao Z, Cea C, Gelinas JN, et al. Translational neuroelectronics. 2020;1909165(29):1–31. 10.1002/adfm.201909165.

[CR34] Johnson I, Liu H. A study on factors affecting the degradation of magnesium and a magnesium-yttrium alloy for biomedical applications. PLoS One. 2013;8(6). 10.1371/journal.pone.0065603. 10.1371/journal.pone.0065603 PMC3683025 23799028

[CR35] Karumbaiah L, Saxena T, Carlson D, Patil K, Patkar R, Gaupp EA, Betancur M, Stanley GB, Carin L, Bellamkonda RV (2013). Relationship between intracortical electrode design and chronic recording function. Biomaterials.

[CR36] Khodagholy D, Doublet T, Quilichini P, Gurfinkel M, Leleux P, Ghestem A, Ismailova E, Hervé T, Sanaur S, Bernard C, Malliaras GG (2013). In vivo recordings of brain activity using organic transistors. Nat Commun.

[CR37] Kim DH, Viventi J, Amsden JJ, Xiao J, Vigeland L, Kim YS, Blanco JA, Panilaitis B, Frechette ES, Contreras D, Kaplan DL, Omenetto FG, Huang Y, Hwang KC, Zakin MR, Litt B, Rogers JA (2010). Dissolvable films of silk fibroin for ultrathin conformal bio-integrated electronics. Nat Mater.

[CR38] Kundu B, Rajkhowa R, Kundu SC, Wang X (2013). Silk fibroin biomaterials for tissue regenerations. Adv Drug Deliv Rev.

[CR39] Kwan MK, Wall EJ, Massie J, Garfin SR (1992). Strain, stress and stretch of peripheral nerve rabbit experiments in vitro and in vivo. Acta Orthop.

[CR40] Lacour SP, Courtine G, Guck J. Materials and technologies for soft implantable neuroprostheses. Nat Rev Mater. 2016;1(10). 10.1038/natrevmats.2016.63.

[CR41] Lecomte A, Castagnola V, Descamps E, Dahan L, Blatché MC, Dinis TM, et al. Silk and PEG as means to stiffen a parylene probe for insertion in the brain: toward a double time-scale tool for local drug delivery. J Micromech Microeng. 2015;25(12). 10.1088/0960-1317/25/12/125003.

[CR42] Lee JW, Lee KY (2017). Dual peptide-presenting hydrogels for controlling the phenotype of PC12 cells. Colloids Surf B Biointerfaces.

[CR43] Li R, Wang L, Kong D, Yin L (2018). Recent progress on biodegradable materials and transient electronics. Bioact Mater.

[CR44] Lissek T. Interfacing neural network components and nucleic acids. Front Bioeng Biotechnol. 2017;5(DEC). 10.3389/fbioe.2017.00053. 10.3389/fbioe.2017.00053 PMC5722975 29255707

[CR45] Lotti F, Ranieri F, Vadalà G, Zollo L, Di Pino G (2017). Invasive intraneural interfaces: foreign body reaction issues. Front Neurosci.

[CR46] Macaya D, Spector M. Injectable hydrogel materials for spinal cord regeneration: a review. Biomed Mater. 2012;7(1). 10.1088/1748-6041/7/1/012001. 10.1088/1748-6041/7/1/012001 22241481

[CR47] Marcombe R, Cai S, Hong W, Zhao X, Lapusta Y, Suo Z, Catoira MC, Fusaro L, Di Francesco D, Ramella M, Boccafoschi F (2019). A theory of constrained swelling of a pH-sensitive hydrogel. J Mater Sci Mater Med.

[CR48] Metallo C, Trimmer BA (2015). Silk coating as a novel delivery system and reversible adhesive for stiffening and shaping flexible probes. J Biol Methods.

[CR49] Mohan T, Kargl R, Tradt KE, Kulterer MR, Braćić M, Hribernik S, Stana-Kleinschek K, Ribitsch V (2015). Antifouling coating of cellulose acetate thin films with polysaccharide multilayers. Carbohydr Polym.

[CR50] Moon HC, Choi H, Kikionis S, Seo J, Youn W, Ioannou E, et al. Fabrication and characterization of neurocompatible ulvan-based layer-by-layer films. 2020;36(39):11610–7. 10.1021/acs.langmuir.0c02173. 10.1021/acs.langmuir.0c02173 32964713

[CR51] Muskovich M, Bettinger CJ (2012). Biomaterials-based electronics: polymers and interfaces for biology and medicine. Adv Healthc Mater.

[CR52] Nair LS, Laurencin CT (2007). Biodegradable polymers as biomaterials. Prog Polym Sci.

[CR53] Nguyen JK, Park DJ, Skousen JL, Hess-Dunning AE, Tyler DJ, Rowan SJ, et al. Mechanically-compliant intracortical implants reduce the neuroinflammatory response. J Neural Eng. 2014;11(5). 10.1088/1741-2560/11/5/056014. 10.1088/1741-2560/11/5/056014 PMC4175058 25125443

[CR54] Olczak KP, McDermott MD, Otto KJ (2019). Electrochemical evaluation of layer-by-layer drug delivery coating for neural interfaces. ACS Appl Bio Mater.

[CR55] Parenteau-Bareil R, Gauvin R, Berthod F (2010). Collagen-based biomaterials for tissue engineering applications. Materials (Basel).

[CR56] Patil AC, Bandla A, Liu YH, Luo B, Thakor NV (2020). Nontransient silk sandwich for soft, conformal bionic links. Mater Today.

[CR57] Patil AC, Xiong Z, Thakor NV (2020). Toward nontransient silk bioelectronics: engineering silk fibroin for bionic links. Small Methods.

[CR58] Pradhan S, Brooks AK, Yadavalli VK (2020). Nature-derived materials for the fabrication of functional biodevices. Mater Today Bio.

[CR59] Redolfi Riva E, Pastoriza-Santos I, Lak A, Pellegrino T, Pérez-Juste J, Mattoli V (2017). Plasmonic/magnetic nanocomposites: gold nanorods-functionalized silica coated magnetic nanoparticles. J Colloid Interface Sci.

[CR60] Redolfi Riva R, Desii A, Sartini S, La Motta C, Mazzolai B, Mattoli V (2013). PMMA/polysaccharides nanofilm loaded with adenosine deaminase inhibitor for targeted anti-inflammatory drug delivery. Langmuir.

[CR61] Redolfi Riva R, Desii A, Sinibaldi E, Ciofani G, Piazza V, Mazzolai B, Mattoli V, Italiano I, Superiore S, Anna S, Riva R, Al ET (2014). Gold nanoshell/polysaccharide nanofilm for controlled laser-assisted tissue thermal ablation. ACS Nano.

[CR62] Renz AF, Reichmuth AM, Stauffer F, Thompson-steckel G, Janos V (2018). A guide towards long-term functional electrodes interfacing neuronal tissue.

[CR63] Righi M, Puleo GL, Tonazzini I, Giudetti G, Cecchini M, Micera S (2018). Peptide-based coatings for flexible implantable neural interfaces. Sci Rep.

[CR64] Rijnbeek EH, Eleveld N, Olthuis W (2018). Update on peripheral nerve electrodes for closed-loop neuroprosthetics. Front Neurosci.

[CR65] Rivnay J, Wang H, Fenno L, Deisseroth K, Malliaras GG. Next-generation probes, particles, and proteins for neural interfacing. Sci Adv. 2017;3(6). 10.1126/sciadv.1601649. 10.1126/sciadv.1601649 PMC5466371 28630894

[CR66] Rochford AE, Carnicer-Lombarte A, Curto VF, Malliaras GG, Barone DG. When bio meets technology: biohybrid neural interfaces. Adv Mater. 2019;(15):1903182. 10.1002/adma.201903182. 10.1002/adma.201903182 31517403

[CR67] Rousche PJ, Pellinen DS, Pivin DP, Williams JC, Vetter RJ, Kipke DR (2001). Flexible polyimide-based intracortical electrode arrays with bioactive capability. IEEE Trans Biomed Eng.

[CR68] Sebaa MA, Dhillon S, Liu H (2013). Electrochemical deposition and evaluation of electrically conductive polymer coating on biodegradable magnesium implants for neural applications. J Mater Sci Mater Med.

[CR69] Shan D, Ma C, Yang J (2019). Enabling biodegradable functional biomaterials for the management of neurological disorders. Adv Drug Deliv Rev.

[CR70] Shanmuganathan K, Capadona JR, Rowan SJ, Weder C (2010). Bio-inspired mechanically-adaptive nanocomposites derived from cotton cellulose whiskers. J Mater Chem.

[CR71] Shen W, Karumbaiah L, Liu X, Saxena T, Chen S, Patkar R, Bellamkonda RV, Allen MG (2015). Extracellular matrix-based intracortical microelectrodes: toward a microfabricated neural interface based on natural materials. Microsyst Nanoeng.

[CR72] Sies H, Sthal W, Sundquist AR (1992). Antioxidant functions of vitamins: vitamins E and C, beta-carotene, and other carotenoids. Ann N Y Acad Sci.

[CR73] Silva JM, Reis RL, Mano JF. Biomimetic extracellular environment based on natural origin polyelectrolyte multilayers. 2016;32(32):4308–42. 10.1002/smll.201601355. 10.1002/smll.201601355 27435905

[CR74] Subbaroyan J, Martin DC, Kipke DR (2005). A finite-element model of the mechanical effects of implantable microelectrodes in the cerebral cortex. J Neural Eng.

[CR75] Tang-Schomer MD, Hu X, Hronik-Tupaj M, Tien LW, Whalen MJ, Omenetto FG, Kaplan DL (2014). Film-based implants for supporting neuron-electrode integrated interfaces for the brain. Adv Funct Mater.

[CR76] Tien LW, Wu F, Tang-Schomer MD, Yoon E, Omenetto FG, Kaplan DL (2013). Silk as a multifunctional biomaterial substrate for reduced glial scarring around brain-penetrating electrodes. Adv Funct Mater.

[CR77] Vitale F, Shen W, Driscoll N, Burrell JC, Richardson AG, Adewole O, Murphy B, Ananthakrishnan A, Oh H, Wang T, Lucas TH, Kacy Cullen D, Allen MG, Litt B (2018). Biomimetic extracellular matrix coatings improve the chronic biocompatibility of microfabricated subdural microelectrode arrays. PLoS One.

[CR78] Wang M, Mi G, Shi D, Bassous N, Hickey D, Webster TJ (2018). Nanotechnology and nanomaterials for improving neural interfaces. Adv Funct Mater.

[CR79] Wellman SM, Eles JR, Ludwig KA, Seymour JP, Michelson NJ, McFadden WE, Vazquez AL, Kozai TDY (2018). A materials roadmap to functional neural Interface design. Adv Funct Mater.

[CR80] Wiraja C, Zhu Y, Lio DCS, Yeo DC, Xie M, Fang W, Li Q, Zheng M, Van Steensel M, Wang L, Fan C, Xu C (2019). Framework nucleic acids as programmable carrier for transdermal drug delivery. Nat Commun.

[CR81] Woods GA, Rommelfanger NJ, Hong G (2020). Review bioinspired materials for in vivo bioelectronic neural interfaces. Matter.

[CR82] Wurth S, Capogrosso M, Raspopovic S, Gandar J, Federici G, Kinany N, Cutrone A, Piersigilli A, Pavlova N, Guiet R, Taverni G, Rigosa J, Shkorbatova P, Navarro X, Barraud Q, Courtine G, Micera S (2017). Long-term usability and bio-integration of polyimide-based intra-neural stimulating electrodes. Biomaterials.

[CR83] Yakuphanoglu F, Aydin ME, Kiliçoǧlu T (2006). Photovoltaic properties of Au/β-carotene/n-Si organic solar cells. J Phys Chem B.

[CR84] Yang J, Du M, Wang L, Li S, Wang G, Yang X, Zhang L, Fang Y, Zheng W, Yang G, Jiang X (2018). Bacterial cellulose as a ssupersoft neural interfacing substrate [research-article]. ACS Appl Mater Interfaces.

[CR85] Yu Y, Shen M, Song Q, Xie J (2018). Biological activities and pharmaceutical applications of polysaccharide from natural resources: a review. Carbohydr Polym.

[CR86] Yuk H, Lu B, Zhao X (2019). Hydrogel bioelectronics. Chem Soc Rev.

[CR87] Zhang C, Wen TH, Razak KA, Lin J, Xu C, Seo C, Villafana E, Jimenez H, Liu H (2020). Magnesium-based biodegradable microelectrodes for neural recording. Mater Sci Eng C.

[CR88] Zhang H, Chiao M (2015). Anti-fouling coatings of poly (dimethylsiloxane) devices for biological and biomedical applications. J Med Biol Eng.

[CR89] Zhang S, Xing M, Li B. Biomimetic layer-by-layer self-assembly of nanofilms, nanocoatings, and 3D scaffolds for tissue engineering. Int J Mol Sci. 2018;19(6):1641. 10.3390/ijms19061641. 10.3390/ijms19061641 PMC6032323 29865178

[CR90] Zhao YH, Niu CM, Shi JQ, Wang YY, Yang YM, Wang HB (2018). Novel conductive polypyrrole/silk fibroin scaffold for neural tissue repair. Neural Regen Res.

